# Meta-analysis on resected pancreatic cancer: a comparison between adjuvant treatments and gemcitabine alone

**DOI:** 10.1186/s12885-018-4948-7

**Published:** 2018-10-23

**Authors:** Hua Chen, Ruizhi He, Xiuhui Shi, Min Zhou, Chunle Zhao, Hang Zhang, Renyi Qin

**Affiliations:** Department of Biliary-Pancreatic Surgery, Affiliated Tongji Hospital, Tongji Medical College, Huazhong University of Science and Technology, Wuhan, 430030 China

**Keywords:** Pancreatic cancer, Gemcitabine, Chemotherapy, Meta-analysis

## Abstract

**Background:**

Pancreatic cancer is a highly malignant tumor with a poor prognosis. Chemotherapy such as gemcitabine is still an important treatment. Gemcitabine (Gem) may prolong survival time and delay the development of recurrent disease after complete resection of pancreatic cancer. Currently, some control studies have been performed between certain drugs and gemcitabine monotherapy after pancreatic cancer surgery, but the outcomes were uncertain. Here, we implemented meta-analysis to compare the efficacy between adjuvant treatments and gemcitabine monotherapy in patients with resected pancreatic cancer.

**Methods:**

PubMed, Embase and the Central Registry of Controlled Trials of the Cochrane Library searches were undertaken to identify randomized controlled trials (RCTs). Date of search ranged from January 1997 to December 2017. The meta-analysis included six RCTs. The major endpoints involved overall survival (OS), disease-free survival/progress free survival/relapse-free survival (DFS/PFS/RFS) and grade 3–4 toxicity.

**Results:**

Pooled meta-analytic estimates were derived using random-effects model. Subgroup analysis used fixed-effects model. The outcome showed that there was no difference in OS (hazard ratio (HR), 0.87; 95% CI, 0.70–1.07; *P* = 0.19) and DFS (HR, 0.85; 95% CI, 0.71–1.02; *P* = 0.08) between the adjuvant treatments group (fluorouracil+folinic acid, S-1, gemcitabine+capecitabine, gemcitabine+erlotinib and gemcitabine+uracil/tegafur) and Gem monotherapy group. However, the subgroup analysis showed that only S-1 chemotherapy, which is an oral fluoropyrimidine agent containing tegafur, gimeracil and oteracil, was significant in OS (HR, 0.59; 95% CI, 0.46–0.74; *P* < 0.0001) and DFS (HR, 0.63; 95% CI, 0.52–0.75; *P* < 0.00001) compared with Gem alone. Toxicity analysis showed there was an increased incidence of grade 3/4 diarrhea (risk ratio (RR), 5.11; 95%CI, 3.24–8.05; *P* < 0.00001) and decreased incidence of grade 3/4 leucopenia (RR, 0.55; 95%CI, 0.31–0.98; *P* = 0.04), thrombocytopenia (RR, 0.61; 95%CI, 0.39–0.97; *P* = 0.04) in adjuvant treatments group. Neutropenia (RR, 0.69; 95%CI, 0.36–1.29; *P* = 0.24) and fatigue (RR, 1.29; 95%CI, 0.95–1.77; *P* = 0.11) for patients between the two groups were not significantly different.

**Conclusions:**

In our meta-analysis, a significant survival benefit is only observed in the S-1 regimen, but the results are yet to be determined. Optimal cytotoxicity or targeted drug regimens need further validation in clinical trials in the future.

**Electronic supplementary material:**

The online version of this article (10.1186/s12885-018-4948-7) contains supplementary material, which is available to authorized users.

## Background

In contrast to the steady increase in survival observed for most cancer types, advances have been slow for pancreatic cancers. More than one-half of cases are diagnosed at a distant stage, for which the 5-year survival rates is 3% [1]. Only a small percentage of patients, approximately 10–15%, have a chance of surgical resection [2–4]. However, the postoperative recurrence rate is high, with approximately 75–92% of patients relapsed [5, 6]. Masato et al. [7] reported that 80% local retroperitoneal recurrence, 66% hepatic metastasis, 53% peritoneal dissemination, 47% lymph node recurrence was discovered in postmortem examinations and 87% recurrence, 53% hepatic metastases in antemortem studies. The median survival after resection of pancreatic cancer remains in the range of 11–20 months and is associated with a 5-year survival rate of 7–25% [8, 9]. More radical resection procedures or extensive lymphadenectomy have not improved the course of disease [8]. Accordingly, adjuvant therapy appears to be very important in order to reduce recurrence and prolong survival after surgery.

The main adjuvant therapy after resection of pancreatic adenocarcinoma included chemotherapy and chemoradiation. Chemoradiation (moderate dose radiation with fluorouracil) has been the standard practice in the U.S. since the study (GITSG 9173) was conducted by the Gastrointestinal Tumor Study Group [10]. The results of some studies also support the survival benefit of chemoradiotherapy for patients with resected pancreatic cancer [11, 12]. But other researches have reached the opposite conclusion [13, 14]. The European Study Group for Pancreatic Cancer (ESPAC-1) took on a head-to-head comparison trial and found a lower median survival for chemoradiation,(15.9 months versus 17.9 months for patients who did not receive chemoradiation) and the median time to recurrence was 10.7 months among patients who received chemoradiotherapy and 15.2 months among those who did not receive chemoradiotherapy [15]. This resulted in far less use of adjuvant chemoradiation in Europe [16]. Chemotherapy mainly includes gemcitabine, fluorouracil, FOLFIRINOX (oxaliplatin, irinotecan, fluorouracil, and leucovorin), and gemcitabine in combination with other drugs. At present, most pancreatologists believe that FOLFIRINOX is superior to gemcitabine. But gemcitabine is still an important chemotherapy drug. Previous clinical trials have demonstrated that postoperative chemotherapy with gemcitabine may prolong survival time and significantly delay the development of recurrent disease after complete resection of pancreatic cancer [5, 17]. Furthermore, some control studies have also been performed between certain drugs and gemcitabine monotherapy after pancreatic cancer surgery, but the outcomes were uncertain. Here, we performed a systematic review and quantitative meta-analysis to assess the role of adjuvant treatments compared with gemcitabine alone after macroscopically complete resection of pancreatic cancer.

## Methods

### Literature search

We retrieved literature with PubMed, Embase and the Central Registry of Controlled Trials of the Cochrane Library and selected studies presented between January 1997, at the time of gemcitabine treatment introduction, and December 2017. The search was performed using the following terms: “pancreatic cancer”, “gemcitabine”, “chemotherapy” and “randomized controlled trial”.

### Inclusion and exclusion criteria

Trials were included in this meta-analysis if they met the following criteria: (1) Patients were required to have histologically proved pancreatic exocrine cancer. In each trial, patients underwent surgery with curative intent (R0 or R1 resection, negative or positive nodal status.R0 resection was defined as no tumor within 1 mm of margin); (2) The treatment group received adjuvant treatments with or without Gem, and the control group received Gem alone; (3) Data contained overall survival (OS) and hazard ratio(HR), disease-free survival / progress free survival / relapse-free survival (DFS/PFS/RFS) and hazard ratio(HR), grade 3–4 toxicity, or OS and DFS curves; (4) This analysis only included RCTs which should be prospective, properly randomized.

Trials were excluded if they met any of the following criteria: (1) Patients received chemotherapy, radiotherapy and other antitumor therapy prior to the study entry. (2) Not RCTs such as case reports, reviews and conference reports; (3) duplicate publications.

### Data collection and analysis

Two investigators (Min Zhou, Chunle Zhao) independently evaluated the abstracts identified from the database. If one of the investigators concluded an abstract was eligible, the full manuscript was retrieved and reviewed in detail by both investigators. If full text of a study could not be obtained, it would be abandoned because of no detailed data to conduct statistical analysis. If the same study appeared on different publications, the one with the latest data was chosen. Methodologic quality of the trials was assessed using Jadad scale [18]. The following information was extracted from each trial: the author, year of publication, number of patients, chemotherapy regimen, OS, DFS/PFS/RFS, grade 3–4 haematological, non-haematological, performance status, etc. Toxicity profiles were reported according to the WHO’s criteria or National Cancer Institute Common Terminology Criteria for Adverse Events (version 3.0).

### Statistical analysis

The primary endpoint was OS after randomization. The other end points were DFS/PFS/RFS and adverse effects. All variables were defined as dichotomous data. We standardized the therapeutic results by obtaining the HR between the adjuvant treatments group and the Gem group. When HR was not reported we estimated it from summary statistics with the method described by Tierney and colleagues [19]. Adverse effects were assessed by RR. Publication bias was investigated by visual inspection of funnel plots. Cochrane’s Q-test and I^2^ statistics were used to assess heterogeneity. For the survival outcome sensitivity analysis was performed to assess the impact of studies with higher risk for bias. A two-tailed *p* value of less than 0.05 was considered statistically significant. All analyses were performed strictly with RevMan software (version 5.3, Cochrane).

## Results

### Trial flow of trials selection

**Fig. 1:**
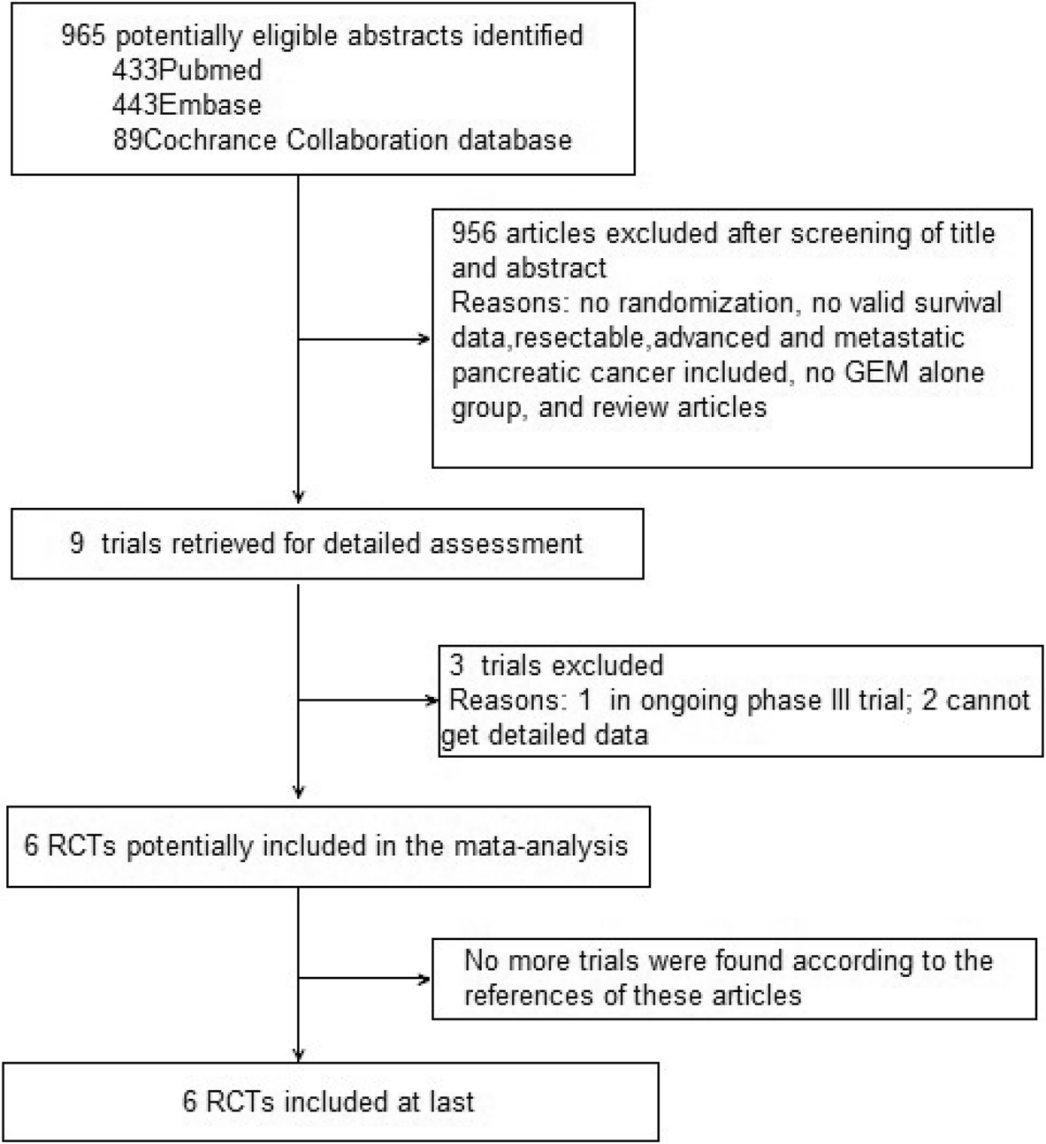
Trial flow of trials selection

The flow chart of this study is shown in Fig. 1. Of the nine trials, three trials are excluded because one reported by Tempero et al. [20] is ongoing trial, the other two by Sinn et al. [21] and Yoshitomi et al. [22] can not obtained detailed data. Both investigators finally agreed to include 6 RCTs in the meta-analysis.

### Characteristics of included trials

These randomized controlled studies are summarized in Additional files 1, 2 and 3. All trials qualities were considered high, with a score of 3 in Jadad scale. Of the six trials, two were randomized phase II trials [23, 24] and the others were randomized phase III trials [25–28]. This meta-analysis evaluated 2787 patients in six randomized trials, of whom 1387 patients were included into the Gem alone arm and 1400 patients into other adjuvant treatments arm. In all six trials, gemcitabine was given 1000 mg/m^2^ once a week for 3 of every 4 weeks. OS and HR with 95% CI, DFS/PFS/RFS and HR with 95% CI were recorded in most of the trials. Grade 3–4 toxicity was recorded in all trials.

Baseline characteristics of the individual trials including gender, performance status (ECOG performance status 0 or Karnofsky performance status (KPS) 60–100%), stage IV of Tumor, pathological resection margin and lymph nodes are indicated in Additional file 1. The distribution of baseline patient characteristics within the respective six trials was found to be homogeneous. However, patients recruited into the study in CONKO-005 were provided with R0 resection.

### Overall survival and sensitivity analysis

The overall survival of six trials are summarized in Additional file 2. The result of the test for heterogeneity of the therapeutic effect was significant (*P* = 0.0006; I^2^ = 77%). Therefore, we selected random effect model. There was not significant in HRs of OS for the adjuvant treatments arm compared with Gem alone arm (HR, 0.87; 95% CI, 0.70–1.07; *P* = 0.19). A sensitivity analysis was performed by excluding JASPAC 01 as it show a best survival benefit (*p* < 0.0001) in the adjuvant treatments group and was therefore considered as an outlier. The meta-analysis of the five remaining studies confirmed no difference in OS between the two arms (HR, 0.96; 95% CI, 0.88–1.06; *P* = 0.44).

### Disease-free survival and sensitivity analysis

The disease-free survival of six trials are summarized in Additional file 2. The result of the test for heterogeneity of the therapeutic effect was significant (*P* = 0.0007; I^2^ = 77%). The outcome of random effect model was not significant in HR of DFS for the adjuvant treatments compared with Gem alone (HR, 0.85; 95% CI, 0.71–1.02; *P* = 0.08). The meta-analysis of the five remaining studies (excluding JASPAC 01) also confirmed no difference in DFS between the two arms (HR, 0.92; 95% CI, 0.80–1.06; *P* = 0.25).

### Subgroup analysis

According to the adjuvant treatments protocol, we divided the trial into three groups such as group1: Fluorouracil+folinic acid (FU + FA) treatment, group 2: S-1 (an oral fluoropyrimidine) treatments and group 3: Gem combined treatments.

**Fig. 2:**
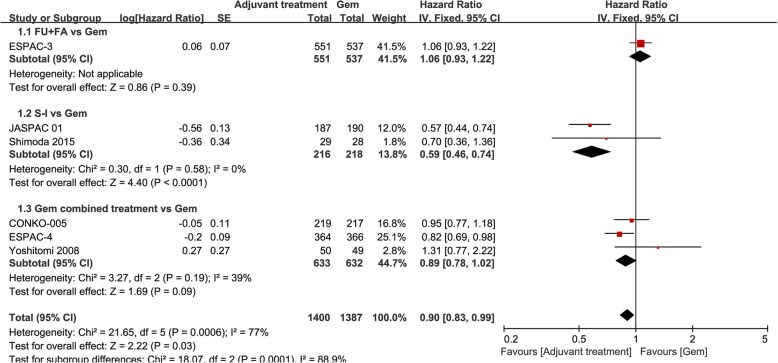
Subgroup analyses of HR of OS for adjuvant treatments vs Gem alone

Figure 2 shows the subgroup analyses of HR of OS. The heterogeneity of subgroup was not significant, which was showed in group 2 (*P* = 0.58; I^2^ = 0%) and group3 (*P* = 0.19; I^2^ = 39%). So we used fixed effect mode. There was not significant in HR of OS for Gem combined treatments group (HR, 0.89; 95% CI, 0.78–1.02; *P* = 0.09) and FU + FA group (HR, 1.06; 95% CI, 0.93–1.22; *P* = 0.39). But for the S-1 group, it was significant (HR, 0.59; 95% CI, 0.46–0.74; *P* < 0.0001).

**Fig. 3:**
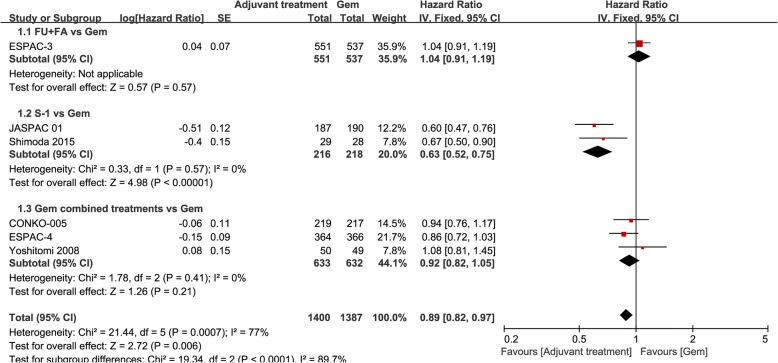
Subgroup analyses of HR of DFS for adjuvant treatments vs Gem alone

Figure 3 shows the subgroup analyses of HR of DFS. The heterogeneity of subgroup was not significant, which was showed group 2 (*P* = 0.57; I^2^ = 0%) and group 3 (*P* = 0.41; I^2^ = 0%). There was not significant in HR of DFS for Gem combined treatments group compared with Gem alone (HR, 0.92; 95% CI, 0.82–1.05; *P* = 0.21) and for FU + FA group (HR, 1.04; 95% CI, 0.91–1.19; *P* = 0.57). But for S-1 group, it was significant (HR, 0.63; 95% CI, 0.52–0.75; *P* < 0.00001).

### Toxic effects of chemotherapy

Six trials reported the incidence of grade 3/4 leucopenia and thrombocytopenia [23–28], five trials reported the incidence of grade 3/4 neutropenia [23, 25–28] and four trials reported the incidence of grade 3/4 anaemia [23, 24, 26, 27]. Five trials reported the incidence of grade 3/4 diarrhea [23, 25–28] and four trials reported the incidence of grade 3/4 fatigue and nausea [25–28].

Grade 3–4 toxicity of subgroup was calculated using method for dichotomous data (RR, 95% CI), (Additional file 3). The pooled results of the meta-analysis revealed an increased incidence of grade 3/4 diarrhea (RR, 5.11; 95%CI, 3.24–8.05; *P* < 0.00001) and decreased incidence of grade 3/4 leucopenia (RR, 0.55; 95%CI, 0.31–0.98; *P* = 0.04), thrombocytopenia (RR, 0.61; 95%CI, 0.39–0.97; *P* = 0.04) in adjuvant treatments group. Neutropenia (RR, 0.69; 95%CI, 0.36–1.29; *P* = 0.24) and fatigue (RR, 1.29; 95%CI, 0.95–1.77; *P* = 0.11) for patients between the two groups was not significantly different.

### Assessment for publication bias

Additional files 4 and 5 represent funnel plots that test for publication bias. Funnel plots for OS (Additional file 4) and DFS (Additional file 5) supported the lack of evidence for publication bias.

## Discussion

For pancreatic cancer treatment, surgery is preferred for best survival [4, 29]. However, the prognosis of the patient remains poor even after curative surgery owing to the high recurrence rate. Since 1996, gemcitabine has become the cornerstone for the treatment of pancreatic cancer. Burris et al. [30] further demonstrated that gemcitabine had a modest survival advantage over treatment with 5-FU in patients with advanced pancreatic cancer. The median survival duration of patients treated with gemcitabine was 5.65 months, compared with 4.41 months for patients treated with 5-FU. The CONKO-001 trial [31] also showed that the median DFS was 13.4 months in the gemcitabine-treated group and 6.7 months in the observation group after radical pancreatic cancer resection (HR, 0.55; 95%CI, 0.44–0.69; *P* < 0.001). Patients randomized to adjuvant gemcitabine therapy had longer OS than those randomized to observation alone (HR, 0.76; 95%CI, 0.61–0.95; *P* = 0.01), with 5-year OS of 20.7% vs 10.4%, respectively, and 10-year OS of 12.2% vs 7.7%. Furthermore, some clinical trials have aimed at assessing the potential superiority of adjuvant chemotherapy over single-agent gemcitabine in resected pancreatic cancer, but the results were not determinate.

In this study, we evaluated six randomized controlled trials comparing adjuvant treatments with gemcitabine monotherapy in first-line treatment of patients undergoing pancreatectomy. Our pooled analysis revealed the overall clinical efficacy of adjuvant chemotherapy was not superior to that of gemcitabine monotherapy in OS (HR, 0.87; 95% CI, 0.70–1.07; *P* = 0.19) and DFS (HR, 0.85; 95% CI, 0.71–1.02; *P* = 0.08). The incidence of adverse events in adjuvant treatments group were increased in grade 3/4 diarrhea and decreased in grade 3/4 leucopenia, thrombocytopenia compared with gemcitabine alone. Although our analysis did not show that adjuvant therapy was superior to gemcitabine monotherapy for resected pancreatic cancer, further stratification analysis was needed given the large heterogeneity of the pooled results.

We next analyzed the clinical efficacy of FU + FA regimen, S-1 regimen and Gem combined regimen respectively, and found that only patients receiving S-1 treatment had a benefit compared with patients receiving gemcitabine alone.

Toxicity of subgroup in S-1 regimen showed the lower incidence of leucopenia, thrombocytopenia, neutropenia and higher diarrhea. Pooled analysis of subgroup analysis showed that S-1 chemotherapy had a significant OS benefit (HR, 0.59; 95% CI, 0.46–0.74; *P* < 0.0001) and DFS benefit (HR, 0.63; 95% CI, 0.52–0.75; *P* < 0.00001). But it should be pointed out that this was only included the results of two trials (*n* = 434). Most patients in the JASPAC 01 [27] study had stage II disease, whereas the majority in another study had stages III and IV [23]. The JASPAC 01 study was only enrolled with patients of Asians. At the same dose, Asians had lower toxic reaction to S-1 than Europeans due to different metabolism. Furthermore, the CAP-002 study [22] also reported that S-1and Gem+S-1(GS) provided similar efficacy to Gem as the adjuvant chemotherapy for resected pancreatic cancer. Two year DFS rate was 24.2%, 28.1% and 34.4% in Gem, S-1 and GS, respectively and the median OS was 21 m in Gem, 26 m in S-1 and 27.9 m in GS.

The ESPAC-1 trial reported that fluorouracil plus folinic acid regimen could improve OS after pancreatic cancer resection, increasing the estimated 2 year and 5 year survival to 40% and 21% compared with 30% and 8.0% for surgery alone [15]. However, the ESPAC-3 [28] trial showed no difference in OS and PFS between the study groups (median OS and 2-year survival rate, 23.0 months and 48.1% in the FU + FA group vs 23.6 months and 49.1% in the Gem group, respectively; *P* = 0.39. median PFS and 2-year survival rate, 14.1 months and 30.7% in the FU + FA group vs 14.3 months and 29.6% in the Gem group, respectively; *P* = 0.53). The outcome of univariate analysis of the ESPAC-3 trial revealed that tumor grade, tumor size, nodal status, resection margin, postoperative CA19–9 levels, performance status, and smoking were independent prognostic factors of OS. But resection margin status was not significant on multivariate analysis, confirming the results of the ESPAC-1 trial that increasingly differentiated tumors, tumor size, and lymph-node status were associated with prognosis. Toxicity analysis of the ESPAC-3 trial showed the lower incidence of leucopenia, thrombocytopenia and higher diarrhea in FU + FA group.

Our subgroup analysis showed that Gem combined therapy did not had a significant OS benefit (HR, 0.89; 95% CI, 0.78–1.02; *P* = 0.09) and DFS benefit (HR, 0.92; 95% CI, 0.82–1.05; *P* = 0.21). It had higher incidence of diarrhea in gemcitabine-based combination therapy group, but leucopenia, thrombocytopenia and neutropenia were no difference between the three groups. In the ESPAC-3 trial [28], the outcome demonstrated that gemcitabine was not superior to fluorouracil plus folinic acid in overall survival for patients with completely resected pancreatic cancer and suggested further comparison of the effects between gemcitabine combined with fluorouracil and folinic acid and gemcitabine monotherapy. Afterward, gemcitabine plus capecitabine (ESPAC-4) [26] trial showed a increase in overall survival, with an estimated 5 year OS of 28.8% (22.9–35.2) compared with 16.3% (10.2–23.7) with Gem and found that prognosis was relationship with postoperative CA19–9 concentrations and pathological margin. However, gemcitabine plus uracil/tegafur had no benefit compared with GEM alone for patients with resected pancreatic cancer. 1-year DFS rate was 50.0% in GU group and 49.0% in the Gem group. Median survival time was 21.2 months and 29.8 months, respectively [24]. Moreover, there were no significant differences in DFS and OS rates between N1, N2 and R0, R1 patients. Although gemcitabine plus erlotinib (GemErlo) had demonstrated a mild survival advantage for advanced pancreatic cancer [32, 33], it did not improve median DFS (GemErlo 11.4 months; Gem 11.4 months) or median overall survival (GemErlo 24.5 months; Gem 26.5 months) in patients with R0 resections in CONKO-005 trial [25]. Similarly, the combination therapy of Gem with sorafenib for 12 months can not improve DFS or OS for R1 resected pancreatic cancer patients in the CONKO-006 trial [21].

At present, some trials demonstrated that the oxaliplatin, irinotecan, fluorouracil, and leucovorin (FOLFIRINOX) and the combination chemotherapy of gemcitabine and nab-paclitaxel regimens had effect on metastatic pancreatic ductal adenocarcinoma (PDAC) [34–36]. Compared with gemcitabine, FOLFIRINOX and nab-paclitaxel plus gemcitabine showed survival advantage [34, 36]. Now, gemcitabine and nab-paclitaxel regimen is being investigated in ongoing phaseIII trial for its efficacy in the adjuvant setting (APACT [NCT01964430: Nab-Paclitaxel and Gemcitabine vs Gemcitabine Alone as Adjuvant Therapy for Patients With Resected Pancreatic Cancer]) [20].

The limitations of this study was the fact that the medicines tested in the trials were different, including chemotherapy drug and molecular targeted drug, which was used alone or in combination. Another limitation was the small number of trials that be included in the study because there were not many of these researches. The third limitation was relatively small number patients of some trials, although the total number of patients included in the meta- analysis was conspicuous.

## Conclusion

In our meta-analysis, a significant survival benefit is only observed in the S-1 regimen, but the results are yet to be determined. Optimal cytotoxicity or targeted drug regimens need further validation in clinical trials in the future. Here, we think that a controlled trial of gemcitabine in combined with S-1 versus FOLFIRINOX or some of the other gents may be a viable option.

## Additional files

Additional file 1: **Table S1.** Characteristics of randomized controlled trials. (XLSX 12 kb)

Additional file 2: **Table S2.** Survival results from randomized trials (adjuvant treatments vs Gem alone). (XLSX 11 kb)

Additional file 3: **Table S3.** The incidence of grade 3/4 adverse events (adjuvant treatments vs Gem alone). (XLSX 11 kb)

Additional file 4: **Figure S1.** Funnel plot for OS for adjuvant treatments vs Gem alone. The outcome supported the lack of evidence for publication bias. (TIF 1836 kb)

Additional file 5: **Figure S2.** Funnel plot for DFS for adjuvant treatments vs Gem alone. The outcome supported the lack of evidence for publication bias. (TIF 1837 kb)

## Abbreviations

Cap: Capecitabine
CI: Confidence interval
DFS: Disease-free survival
Erlo: Erlotinib
FA: Folinic acid
FU: 5-Fluorouracil
Gem: Gemcitabine
GemErlo: Gemcitabine+erlotinib
GU: Gemcitabine+uracil/tegafur
HR: Hazard ratio
KPS: Karnofsky performance status
NA: Data not available
OS: Overall survival
PFS: Progress free survival
RCTs: Randomized controlled trials
RFS: Relapse-free survival
RR: Risk ratio
UFT: Uracil/tegafur

## References

[1] Siegel RL, Miller KD, Jemal A (2018). Cancer statistics, 2018. CA Cancer J Clin.

[2] Beger HG, Rau B, Gansauge F, Poch B, Link KH (2003). Treatment of pancreatic cancer: challenge of the facts. World J Surg.

[3] Cress RD, Yin D, Clarke L, Bold R, Holly EA (2006). Survival among patients with adenocarcinoma of the pancreas: a population-based study (United States). Cancer Causes Control.

[4] Sener SF, Fremgen A, Menck HR, Winchester DP (1999). Pancreatic cancer: a report of treatment and survival trends for 100,313 patients diagnosed from 1985-1995, using the National Cancer Database. J Am Coll Surg.

[5] Oettle H, Post S, Neuhaus P, Gellert K, Langrehr J, Ridwelski K, Schramm H, Fahlke J, Zuelke C, Burkart C (2007). Adjuvant chemotherapy with gemcitabine vs observation in patients undergoing curative-intent resection of pancreatic cancer: a randomized controlled trial. JAMA.

[6] Van den Broeck A, Sergeant G, Ectors N, Van Steenbergen W, Aerts R, Topal B (2009). Patterns of recurrence after curative resection of pancreatic ductal adenocarcinoma. Eur J Surg Oncol.

[7] Kayahara M, Nagakawa T, Ueno K, Ohta T, Takeda T, Miyazaki I (1993). An evaluation of radical resection for pancreatic cancer based on the mode of recurrence as determined by autopsy and diagnostic imaging. Cancer.

[8] Alexakis N, Halloran C, Raraty M, Ghaneh P, Sutton R, Neoptolemos JP (2004). Current standards of surgery for pancreatic cancer. Br J Surg.

[9] Brunner TB, Grabenbauer GG, Meyer T, Golcher H, Sauer R, Hohenberger W (2007). Primary resection versus neoadjuvant chemoradiation followed by resection for locally resectable or potentially resectable pancreatic carcinoma without distant metastasis. A multi-centre prospectively randomised phase II-study of the Interdisciplinary Working Group Gastrointestinal Tumours (AIO, ARO, and CAO). BMC Cancer.

[10] Kalser MH, Ellenberg SS (1985). Pancreatic cancer. Adjuvant combined radiation and chemotherapy following curative resection. Arch Surg.

[11] Regine WF, Winter KA, Abrams RA, Safran H, Hoffman JP, Konski A, Benson AB, Macdonald JS, Kudrimoti MR, Fromm ML (2008). Fluorouracil vs gemcitabine chemotherapy before and after fluorouracil-based chemoradiation following resection of pancreatic adenocarcinoma: a randomized controlled trial. JAMA.

[12] Yeo CJ, Abrams RA, Grochow LB, Sohn TA, Ord SE, Hruban RH, Zahurak ML, Dooley WC, Coleman J, Sauter PK (1997). Pancreaticoduodenectomy for pancreatic adenocarcinoma: postoperative adjuvant chemoradiation improves survival. A prospective, single-institution experience. Ann Surg.

[13] Neoptolemos JP, Dunn JA, Stocken DD, Almond J, Link K, Beger H, Bassi C, Falconi M, Pederzoli P, Dervenis C (2001). Adjuvant chemoradiotherapy and chemotherapy in resectable pancreatic cancer: a randomised controlled trial. Lancet.

[14] Smeenk HG, van Eijck CH, Hop WC, Erdmann J, Tran KC, Debois M, van Cutsem E, van Dekken H, Klinkenbijl JH, Jeekel J (2007). Long-term survival and metastatic pattern of pancreatic and periampullary cancer after adjuvant chemoradiation or observation: long-term results of EORTC trial 40891. Ann Surg.

[15] Neoptolemos JP, Stocken DD, Friess H, Bassi C, Dunn JA, Hickey H, Beger H, Fernandez-Cruz L, Dervenis C, Lacaine F (2004). A randomized trial of chemoradiotherapy and chemotherapy after resection of pancreatic cancer. N Engl J Med.

[16] Twombly R (2008). Adjuvant chemoradiation for pancreatic cancer: few good data, much debate. J Natl Cancer Inst.

[17] Ueno H, Kosuge T, Matsuyama Y, Yamamoto J, Nakao A, Egawa S, Doi R, Monden M, Hatori T, Tanaka M (2009). A randomised phase III trial comparing gemcitabine with surgery-only in patients with resected pancreatic cancer: Japanese study Group of Adjuvant Therapy for pancreatic Cancer. Br J Cancer.

[18] Jadad AR, Moore RA, Carroll D, Jenkinson C, Reynolds DJ, Gavaghan DJ, McQuay HJ (1996). Assessing the quality of reports of randomized clinical trials: is blinding necessary?. Control Clin Trials.

[19] Tierney JF, Stewart LA, Ghersi D, Burdett S, Sydes MR (2007). Practical methods for incorporating summary time-to-event data into meta-analysis. Trials.

[20] Tempero MA, Cardin DB, Biankin A, Goldstein D, Moore M, O’Reilly EM, Philip PA, Riess H, Macarulla T, Yung L (2015). nab-paclitaxel (nab-P) plus gemcitabine (Gem) vs Gem alone as adjuvant treatment for resected pancreatic cancer (PC) in a phase III trial (APACT). J Clin Oncol.

[21] Sinn M, Liersch T, Gellert K, Riess H, Stübs P, Waldschmidt DT, Pelzer U, Stieler J, Striefler JK, Bahra M (2014). LBA18CONKO-006: A randomized double-blinded phase IIB-study of adjuvant therapy with gemcitabine + sorafenib/placebo for patients with R1-resection of pancreatic cancer. Ann Oncol.

[22] Yoshitomi H, Shimizu H, Yoshidome H, Ohtsuka M, Kato A, Furukawa K, Takayashiki T, Kuboki S, Okamura D, Suzuki D (2013). A randomized phase II trial of adjuvant chemotherapy with S-1 versus S-1 and gemcitabine (GS) versus gemcitabine alone (GEM) in patients with resected pancreatic cancer (CAP-002 study). J Clin Oncol.

[23] Shimoda M, Kubota K, Shimizu T, Katoh M (2015). Randomized clinical trial of adjuvant chemotherapy with S-1 versus gemcitabine after pancreatic cancer resection. Br J Surg.

[24] Yoshitomi H, Togawa A, Kimura F, Ito H, Shimizu H, Yoshidome H, Otsuka M, Kato A, Nozawa S, Furukawa K (2008). A randomized phase II trial of adjuvant chemotherapy with uracil/tegafur and gemcitabine versus gemcitabine alone in patients with resected pancreatic cancer. Cancer.

[25] Sinn M, Bahra M, Liersch T, Gellert K, Messmann H, Bechstein W, Waldschmidt D, Jacobasch L, Wilhelm M, Rau BM (2017). CONKO-005: adjuvant chemotherapy with gemcitabine plus erlotinib versus gemcitabine alone in patients after R0 resection of pancreatic cancer: a multicenter randomized phase III trial. J Clin Oncol.

[26] Neoptolemos JP, Palmer DH, Ghaneh P, Psarelli EE, Valle JW, Halloran CM, Faluyi O, O’Reilly DA, Cunningham D, Wadsley J (2017). Comparison of adjuvant gemcitabine and capecitabine with gemcitabine monotherapy in patients with resected pancreatic cancer (ESPAC-4): a multicentre, open-label, randomised, phase 3 trial. Lancet.

[27] Uesaka K, Boku N, Fukutomi A, Okamura Y, Konishi M, Matsumoto I, Kaneoka Y, Shimizu Y, Nakamori S, Sakamoto H (2016). Adjuvant chemotherapy of S-1 versus gemcitabine for resected pancreatic cancer: a phase 3, open-label, randomised, non-inferiority trial (JASPAC 01). Lancet.

[28] Neoptolemos JP, Stocken DD, Bassi C, Ghaneh P, Cunningham D, Goldstein D, Padbury R, Moore MJ, Gallinger S, Mariette C (2010). Adjuvant chemotherapy with fluorouracil plus folinic acid vs gemcitabine following pancreatic cancer resection: a randomized controlled trial. JAMA.

[29] Imamura M, Doi R, Imaizumi T, Funakoshi A, Wakasugi H, Sunamura M, Ogata Y, Hishinuma S, Asano T, Aikou T (2004). A randomized multicenter trial comparing resection and radiochemotherapy for resectable locally invasive pancreatic cancer. Surgery.

[30] Burris HA, Moore MJ, Andersen J, Green MR, Rothenberg ML, Modiano MR, Cripps MC, Portenoy RK, Storniolo AM, Tarassoff P (1997). Improvements in survival and clinical benefit with gemcitabine as first-line therapy for patients with advanced pancreas cancer: a randomized trial. J Clin Oncol.

[31] Oettle H, Neuhaus P, Hochhaus A, Hartmann JT, Gellert K, Ridwelski K, Niedergethmann M, Zulke C, Fahlke J, Arning MB (2013). Adjuvant chemotherapy with gemcitabine and long-term outcomes among patients with resected pancreatic cancer: the CONKO-001 randomized trial. JAMA.

[32] Gresham GK, Wells GA, Gill S, Cameron C, Jonker DJ (2014). Chemotherapy regimens for advanced pancreatic cancer: a systematic review and network meta-analysis. BMC Cancer.

[33] Moore MJ, Goldstein D, Hamm J, Figer A, Hecht JR, Gallinger S, Au HJ, Murawa P, Walde D, Wolff RA (2007). Erlotinib plus gemcitabine compared with gemcitabine alone in patients with advanced pancreatic cancer: a phase III trial of the National Cancer Institute of Canada Clinical Trials Group. J Clin Oncol.

[34] Conroy T, Desseigne F, Ychou M, Bouche O, Guimbaud R, Becouarn Y, Adenis A, Raoul JL, Gourgou-Bourgade S, de la Fouchardiere C (2011). FOLFIRINOX versus gemcitabine for metastatic pancreatic cancer. N Engl J Med.

[35] Von Hoff DD, Ramanathan RK, Borad MJ, Laheru DA, Smith LS, Wood TE, Korn RL, Desai N, Trieu V, Iglesias JL (2011). Gemcitabine plus nab-paclitaxel is an active regimen in patients with advanced pancreatic cancer: a phase I/II trial. J Clin Oncol.

[36] Von Hoff DD, Ervin T, Arena FP, Chiorean EG, Infante J, Moore M, Seay T, Tjulandin SA, Ma WW, Saleh MN (2013). Increased survival in pancreatic cancer with nab-paclitaxel plus gemcitabine. N Engl J Med.

