# Supplementary figures and images for: Meta-analysis on resected pancreatic cancer: a comparison between adjuvant treatments and gemcitabine alone
Source: BMC Cancer. 2018 Oct 23;18:1034. doi: 10.1186/s12885-018-4948-7 (PMC6199735; doi:10.1186/s12885-018-4948-7)

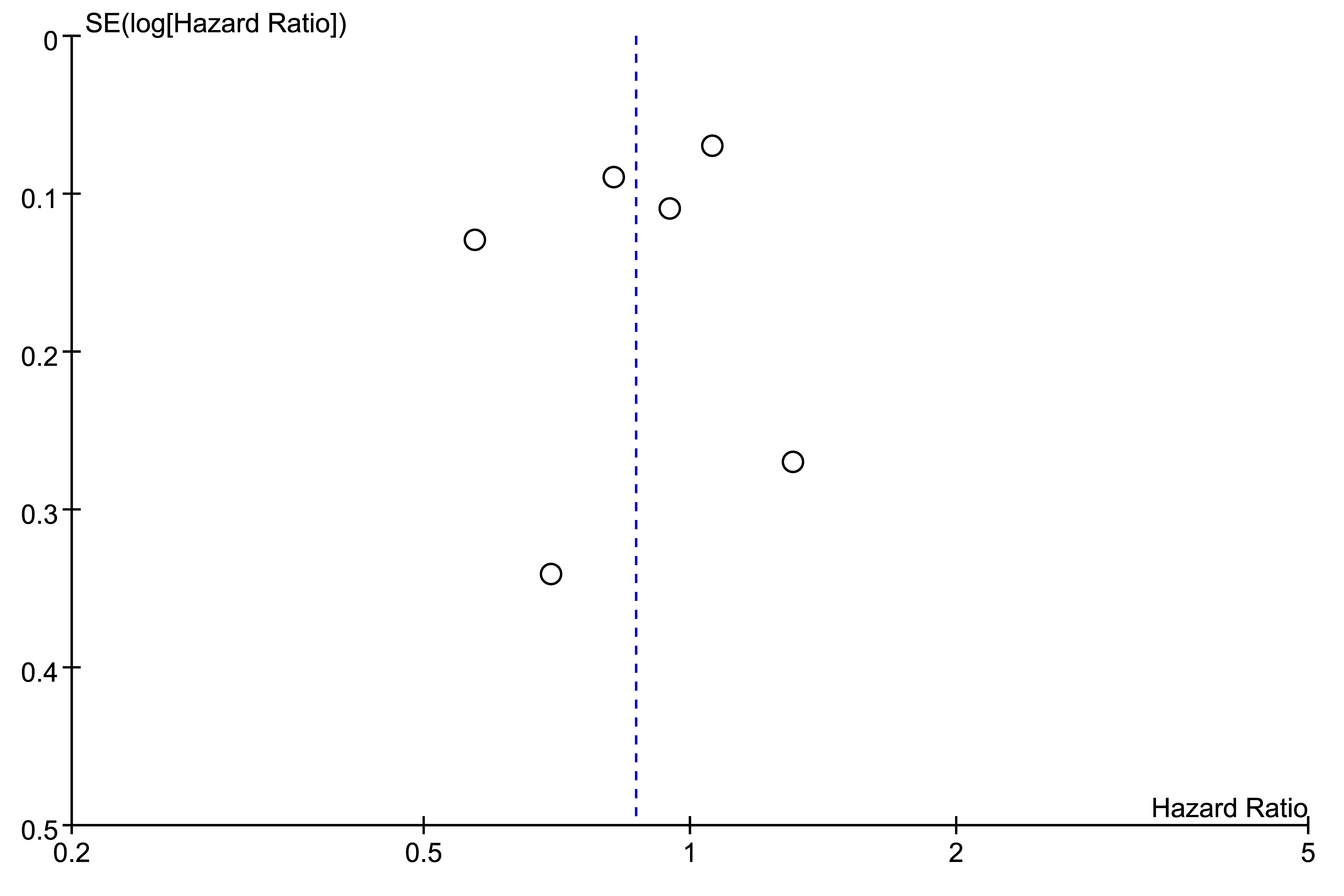

Supplement: Supplementary file 4 — Figure S1. Funnel plot for OS for adjuvant treatments vs Gem alone. The outcome supported the lack of evidence for publication bias. (TIF 1836 kb) [file 12885_2018_4948_MOESM4_ESM.tif]

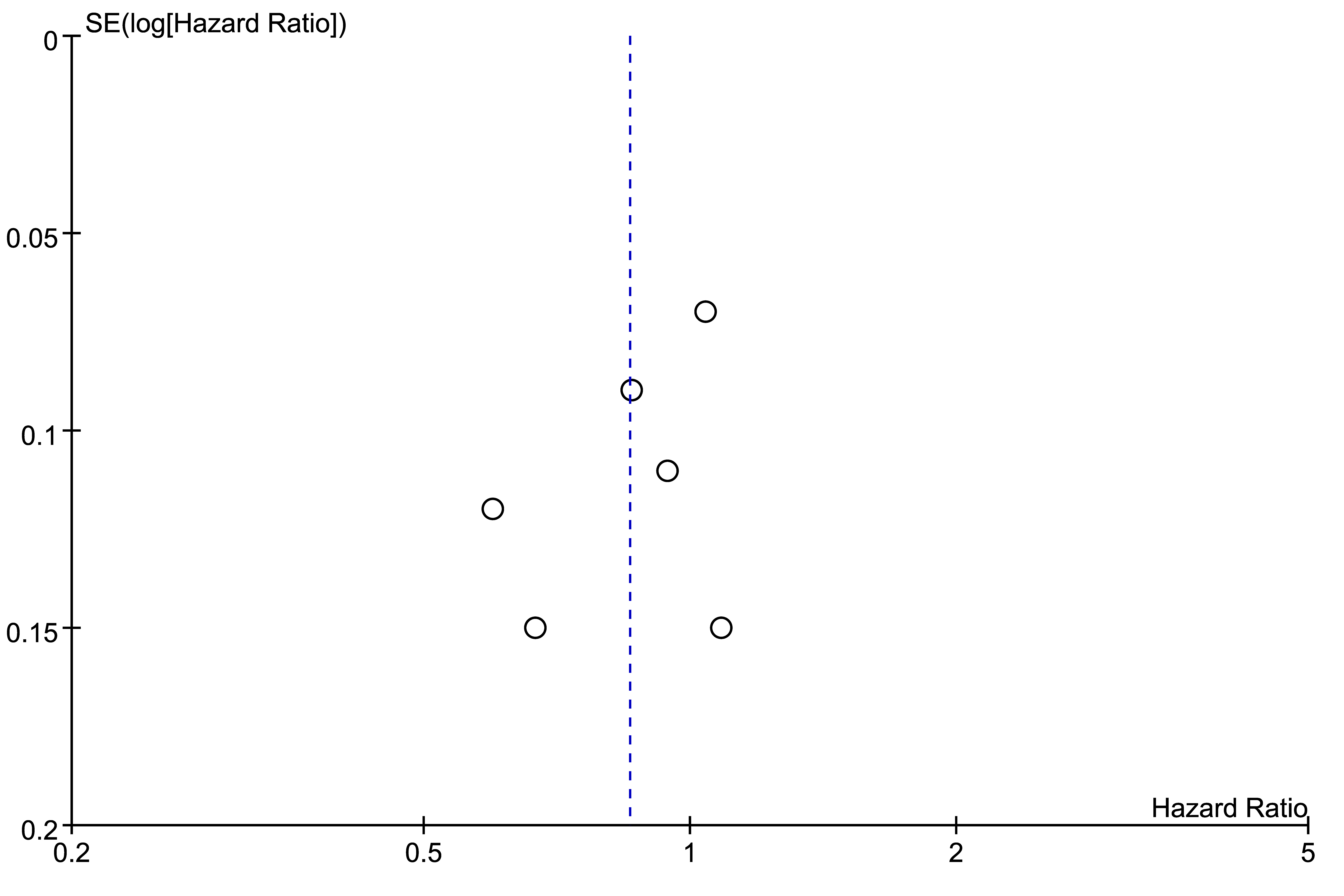

Supplement: Supplementary file 5 — Figure S2. Funnel plot for DFS for adjuvant treatments vs Gem alone. The outcome supported the lack of evidence for publication bias. (TIF 1837 kb) [file 12885_2018_4948_MOESM5_ESM.tif]
